# Arbuscular Mycorrhizal Fungi Induced Plant Resistance against Fusarium Wilt in Jasmonate Biosynthesis Defective Mutant and Wild Type of Tomato

**DOI:** 10.3390/jof8050422

**Published:** 2022-04-20

**Authors:** Haixi Wang, Zhipeng Hao, Xin Zhang, Wei Xie, Baodong Chen

**Affiliations:** 1 State Key Laboratory of Urban and Regional Ecology, Research Center for Eco-Environmental Sciences, Chinese Academy of Sciences, Beijing 100085, China; wanghaixi98@163.com (H.W.); xinzhang@rcees.ac.cn (X.Z.); xieweibisheng@yeah.net (W.X.); bdchen@rcees.ac.cn (B.C.); 2 University of Chinese Academy of Sciences, Beijing 100049, China

**Keywords:** arbuscular mycorrhizal fungi, *Fusarium oxysporum*, mycorrhiza induced resistance, genotype-specific trait, jasmonate signaling pathway

## Abstract

Arbuscular mycorrhizal (AM) fungi can form mutual symbiotic associations with most terrestrial plants and improve the resistance of host plants against pathogens. However, the bioprotection provided by AM fungi can depend on the host–fungus combinations. In this study, we unraveled the effects of pre-inoculation with AM fungus *Rhizophagus irregularis* on plant resistance against the hemibiotrophic fungal pathogen *Fusarium oxysporum* in jasmonate (JA) biosynthesis mutant tomato, *suppressor of prosystemin-mediated responses8* (*spr8*) and the wild type Castlemart (CM). Results showed that *R. irregularis* colonization in CM plants significantly decreased the disease index, which was not observed in *spr8* plants, suggesting that the disease protection of AM fungi was a plant-genotype-specific trait. Inoculation with *R. irregularis* significantly increased the shoot dry weight of CM plants when infected with *F. oxysporum*, with increased plant P content and net photosynthetic rate. Induced expression of the JA synthesis genes, including allene oxide cyclase gene (*AOC*) and lipoxygenase D gene (*LOXD*), and increased activities of polyphenol oxidase (PPO) and phenylalanine ammonia lyase (PAL) were recorded in mycorrhizal CM plants infected with *F. oxysporum*, but not in *spr8* plants. Thus, mycorrhiza-induced resistance (MIR) to fungal pathogen in tomato was highly relevant to the JA signaling pathway.

## 1. Introduction

Tomato (*Solanum lycopersicum* Mill.) is a vegetable crop with the greatest production worldwide owing to its unique flavor, rich nutrition and good taste [1]. However, soilborne pathogens remain a serious threat to the tomato industry [2,3]. *Fusarium oxysporum* f. sp. *lycopersici* is one of the primary causes of tomato yield losses in agricultural systems [4,5]. *F. oxysporum* intensively colonizes vascular tissues and interferes with water transport and nutrient absorption, resulting in leaf chlorosis, root necrosis and plant death [6]. Furthermore, *F. oxysporum* can be easily spread by water, soil, wind and plant debris [7]. The potential control strategies against tomato *Fusarium* wilt are chemical agents, soil fumigation, screening for resistant cultivars and biological methods [8,9]. Among them, biological control agents involving antagonistic or competitive microorganisms against pathogens are attracting extensive attention because of their ecological sustainability and environmental friendliness [10,11,12,13].

Arbuscular mycorrhizal (AM) fungi are a group of biotrophic fungi that can form symbiotic associations with most terrestrial plants [14]. This ancient symbiosis has existed for more than 400 million years [15,16]. In AM symbiosis, plants allocate a portion of photosynthates to AM fungi to support fungal growth. AM fungi facilitate plant nutrient and water uptake from soil [17]. Owing to the unique symbiotic mechanism, AM fungi play a critical role in promoting plant growth [18,19] and enhancing the plant resistance to abiotic and biotic stresses [20,21].

AM symbioses can enhance plant resistance against various pathogens, including nematodes, fungi, bacteria and viruses, especially soilborne pathogens [22,23,24,25]. For instance, inoculation with AM fungus *Glomus etunicatum* BEG168 enhanced plant resistance to *F. oxysporum* f. sp. *cuncumerinum*, the causal agent of cucumber (*Cucumis sativus* L.) wilt disease [26]. AM fungal bioprotection against several plant diseases has been described for various plant species; however, the effectiveness of bioprotection provided by AM fungi may depend on the host–fungus combinations. A study on two tomato varieties (Micro-Tom and Missouri) showed that only Missouri benefited from the inoculation with the AM fungus *Rizophagus irregularis* (DAOM 197198) against *Xanthamonas campestris* pv. *vesicatoria*, whereas this protection effect was not observed in Micro-Tom [27].

Mycorrhiza-induced resistance (MIR) is associated with improved plant mineral nutrition, especially phosphorus (P) [28]. Improved plant nutrient contents by AM fungi further increases plant photosynthetic rate, leading to increased plant biomass [29]. MIR is also considered to be a crucial mechanism for disease control [30]. Mycorrhizal colonization in root cortex can elicit specific plant reactions and further activate the plant defense system. Effector proteins and microbe-associated molecular patterns (MAMP) can be recognized by plant receptor protein complexes, leading to the activation of effector-triggered immunity (ETI) or MAMP-triggered immunity (MTI) [31]. Kloppholz et al. [32] showed that the effector molecule secreted protein 7 (SP7) of the AM fungus *Glomus intraradices* promoted the formation of AM symbiosis by suppressing plant immune responses. MTI associated with AM fungi triggers the synthesis of secondary metabolites and the production of reactive oxygen [33,34]. During the initial stages of mycorrhizal colonization, MAMPs from AM fungi generate a transient and weak MTI, which primes the strong defensive responses to pathogens [35]. Increased activities of polyphenol oxidase (PPO) and phenylalanine ammonia lyase (PAL) are recorded in mycorrhizal plants infected by pathogens, but PPO and PAL are unchanged in plants inoculated with only AM fungi [36].

Plant defense responses can be coordinated by several small molecules such as jasmonic acid (JA), salicylic acid (SA) and ethylene (ET), which coordinate the expression of defense-related genes [37,38]. Several studies have proposed that the effectiveness of MIR is due to the quick activation of the JA-dependent signaling pathway [39,40,41], which is a pivot regulator in plant defense against necrotrophs and chewing insects [42]. Minton et al. [39] confirmed the upregulated PPO activity with JA application in *Solanum dulcamara*. Nair et al. [43] showed higher activity of lipoxygenase (LOX), which is involved in JA biosynthesis, in AM plants correlated with higher levels of methyl jasmonate (MeJA). Furthermore, genes involved in JA biosynthesis and response also prove expression upregulation in mycorrhizal plants. However, the SA-dependent pathway regulates plant defense-related responses against (hemi)biotrophic pathogens [44] and the JA and SA pathways are mutually antagonistic [45]. The underlying mechanism of MIR of different tomato genotype against *F. oxysporum* remains highly controversial.

In the present study, we unraveled the bioprotection effects of pre-inoculation with AM fungus *Rhizophagus irregularis* on plant resistance against *F. oxysporum* of the JA biosynthesis mutant tomato, *suppressor of prosystemin-mediated responses8* (*spr8*) [46] and the wild-type Castlemart (CM). The study would enable unequivocal dissection of the potential involvement of the JA signaling pathway in MIR by comparing the expression of allene oxide cyclase (*AOC*) and lipoxygenase D *(LOXD*) genes involving JA biosynthesis and the activity of defense-related marker enzymes.

## 2. Materials and Methods

### 2.1. Plants, Fungi and Growth Substrate

Indeterminate tomato seeds (CM and *spr8*) were obtained from the Institute of Genetics and Developmental Biology, Chinese Academy of Sciences. The seeds were surface sterilized with 10% H_2_O_2_ for 10 min and rinsed with sterile water. The sterilized seeds were germinated on soaked sterile filter papers in darkness at 25 °C.

The AM fungus *R. irregularis* Schenck & Smith (BGC AH01) was obtained from the Beijing Academy of Agriculture and Forestry. The inoculum comprised cultivation medium (a mixture of sterilized zeolite (<3 mm), sand (<2 mm) and sandy loam at a ratio of 7:2:1(*V*:*V*:*V*)), colonized root fragments, fungal spores (67 spores g^−1^) and extraradical hyphae.

*F. oxysporum* was obtained from Fujian Agriculture and Forestry University. *F. oxysporum* was incubated on potato dextrose agar at 25 °C for 7 days [24]. Hyphal tips were then transferred to 100 mL of sterilized potato dextrose broth incubated at 25 °C with shaking (120 rpm) for 7 days. The culture was filtered through three layers of sterile gauze and centrifuged twice (5 min, 3000× *g*) to collect *F. oxysporum* conidia. After removing the supernatant, the conidia pellet was washed with sterile water three times and suspended in sterile water [47]. The concentration was determined using a hemocytometer and adjusted to 10^8^ conidia mL^−1^ [48].

The growth substrate was a 1:1 (*w*:*w*) mixture of sand (<2 mm) and soil. The soil was collected from Yanqing Field Experimental and Demonstration Base, Research Center for Eco-Environmental Sciences, Chinese Academy of Sciences, located in Tangjiapu, Yanqing District, Beijing, China (40°47′ N, 116°34′ E). The soil was passed through a 2 mm sieve and sterilized using γ-radiation (10 MeV electron beam, 20 kGy) before use. The soil was loamy, with an organic matter content of 4.42%, NO_3_^−^-N concentration of 8.3 mg kg^−1^, NH_4_^+^-N concentration of 5.5 mg kg^−1^, available P concentration of 15.0 mg kg^−1^ and pH of 7.7.

### 2.2. Experimental Design and Procedure

The study comprised eight treatments, including two different genotypes (CM, *spr8*) inoculated with (+M) or without (−M) *R. irregularis* and either inoculated with (+F) or free from (−F) *F. oxysporum*. Each treatment comprised nine replicates, giving a total of 72 pots with a completely randomized block design.

For +M treatment, 7% *R. irregularis* inoculum was added to growth substrate and thoroughly mixed before seeding, whereas −M treatment received equivalent sterilized AM inoculum with 5 mL of inoculum filtrate to provide a similar microbiota except for AM fungus [22]. The filtrate was obtained by passing mycorrhizal inoculum in sterilized water (1:4, *w*:*w*) through filter paper (15–20 μm).

*F. oxysporum* was inoculated at 55 days after the potting procedure. Plants were carefully removed from the growth substrate and washed. *F. oxysporum* was applied by dipping the roots for 30 min in a conidial suspension, whereas –F plants were treated with sterilized water [48]. All plants were replanted into newly prepared sterile soil for six days.

The experiment was conducted in a climate chamber with 300 μmol m^−2^ s^−1^ light intensity, 16 h/8 h (light/dark) photoperiod, 25 °C/18 °C (light/dark) and 60% relative humidity. Plants were irrigated daily with deionized water to maintain the growth substrate water content to 75% of the field water capacity.

### 2.3. Net Photosynthetic and Transpiration Rate

Net photosynthetic and transpiration rate were measured using a portable photosynthetic system (Li-6800, LI-COR Biosciences, Lincoln, NE, USA) before harvest. The parameter settings in the leaf chamber were as follows: photosynthetic photon flux density of 800 μmol m^−2^ s^−1^, CO_2_ concentration of 400 μmol mol^−1^ and relative humidity 65%. The third mature leaf from the top of each plant was selected for the measurements.

### 2.4. Disease Assessment

The assessment of *Fusarium* wilt was conducted by visual observation. Disease severity was evaluated according to the following scale: 0, no wilt symptoms; 1, 1–33% of leaves yellowed or wilted; 2, 34–67% of leaves yellowed or wilted; 3, 68–100% of leaves yellowed or wilted; and 4, dead [47]. Disease index (DI) was calculated according to the following formula: DI = [∑(rating score × number of plants rated)/(total number of plants × 4)] × 100 [49].

### 2.5. Plant Harvest

The roots from each pot were removed and washed with deionized water. Shoots and roots were harvested and weighed separately. Part of the root sample from each plant was placed in liquid nitrogen and stored at −80 °C for RNA extraction and enzyme activity determination. Some fresh root samples (~0.5 g) were collected to determine mycorrhizal colonization. The remaining shoot and root samples were dried at 105 °C for 30 min and at 70 °C for 24 h to constant weight for elemental content determination.

### 2.6. Mycorrhizal Colonization

Fresh root samples were cut into 1 cm segments and incubated in 10% KOH at 90 °C for 10–20 min. After acidification with 2% HCl for 5 min, the root segments were stained in trypan blue (0.05%) at 90 °C for 10 min and decolorized in lactic acid glycerin (lactic acid–glycerin–water = 1:1:1) for more than 12 h. Thirty root segments were randomly selected and examined under a microscope [50]. Mycorrhizal colonization rate (%) and arbuscule abundance (%) were calculated using MYCOCALC software [51].

### 2.7. Plant P Concentrations

The dried shoot and root samples were ground with a ball mill and digested in a Microwave Accelerated Reaction System (Mars 5, CEM, Matthews, NC, USA). An inductively coupled plasma optical emission spectrometer (ICP-OES, Prodigy, Teledyne Leeman, Hudson, NH, USA) was used to determine the P concentrations in shoot and root samples.

### 2.8. RNA Extraction and Gene Expression Analysis

Total root RNA was extracted using RNeasy Plant Mini Kit (Qiagen, Dusseldorf, Germany). The extracted RNA was digested with DNase I (Thermo Fisher Scientific Inc., Waltham, MA, USA). cDNA was synthesized using a RevertAid First Strand cDNA Synthesis Kit (Thermo Fisher Scientific Inc., USA). *AOC* and *LOXD* expression were quantified by quantitative real-time PCR using a Bio-Rad CFX96 Optical system (Bio-Rad, Hercules, CA, USA) with SYBR Green I fluorescence (TAKARA Biotechnology Co. Ltd., Japan). The primers for *AOC* (AW624058) were 5′-CTCGGAGATCTTGTCCCCTTT-3′ and 5′-CTCCTTTCTTCTCTTCTTCGTGCT-3′, whereas the primers for *LOXD* (U37840) were 5′-CCGTGGTTGACACATTATCG-3′ and 5′-ACAGCAGTCCGCCCTATTTA-3′ [40]. The quantitative PCR procedure was as follows: an initial denaturation phase at 95 °C for 45 s, followed by 35 cycles at 95 °C for 30 s, 56 °C for 30 s and 72 °C for 60 s. The melting curve was programmed as follows: 70 °C for 10 s and then heated to 95 °C at a rate of 0.5 °C s^−1^. Data were collected continuously. Each sample was technically parallel thrice and each treatment had four biological replicates. The relative gene expression was calculated by the 2^−ΔΔCt^ method [52] and *Ubi3* was used as a reference [53].

### 2.9. PPO and PAL Activities

PPO and PAL activities were quantified following the method of Zhou et al. [54] using Polyphenol Oxidase (PPO) Assay Kits and Phenylalanine Ammonia Lyase (PAL) Assay Kits (Beijing Solarbio Science & Technology Co., Ltd., Beijing, China) according to the manufacturer’s instructions.

### 2.10. Statistical Analysis

Before statistical analysis, all data were checked for normality and variance homogeneity by using the Shapiro–Wilk test and Levene’s test. Mycorrhizal colonization rate, arbuscule abundance and disease index (percentage values) were arcsine (square root (X)) transformed. In case of variance homogeneity, three-way analysis of variance (ANOVA) was performed to test the significance of the treatment effects and the interactions among plant genotypes (G), inoculation with *R. irregularis* (Myc) and inoculation with *F. oxysporum* (Fol). Multi comparisons across all treatments were performed using one-way ANOVA followed by Duncan’s multiple-range test (*p* < 0.05). In the case of heterogeneity of variance, nonparametric Kruskal–Wallis was used to conduct data analysis. All data were analyzed using IBM SPSS Statistics 21.0 (IBM Corp., Armonk, NY, USA).

## 3. Results

### 3.1. Mycorrhizal Colonization

No mycorrhizal colonization was observed in roots not inoculated with *R. irregularis* (Table 1). The mycorrhizal colonization rates of inoculated plants were over 30%, indicating that *R. irregularis* had formed a symbiotic association with both genotypes. The mycorrhizal colonization rate and arbuscule abundance of *Spr8* plants were significantly lower than those of CM plants (*p* < 0.001 **). *F. oxysporum* inoculation generally had no effect on mycorrhizal colonization.

### 3.2. Disease Index

**Figure 1:**
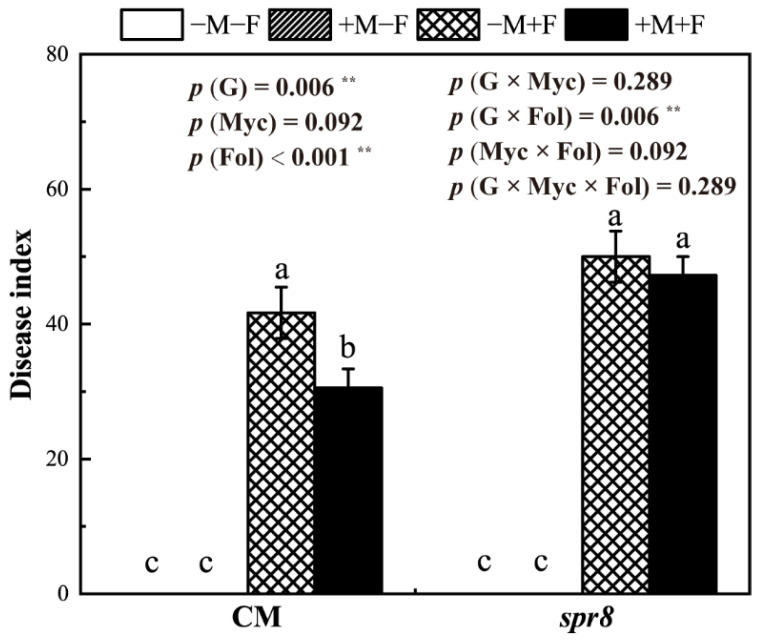
Effects of inoculation with *Rhizophagus irregularis* (Myc) and *Fusarium oxysporum* f. sp. *Lycopersici* inoculation (Fol) on the disease index of CM and *spr8* plants (G). −M and +M represent non-inoculation and inoculation with *Rhizophagus irregularis*, respectively. −F and +F represent non-inoculation and inoculation with *Fusarium oxysporum* f. sp. *Lycopersici*, respectively. Same lowercase letters above the columns indicate non-significant difference (*p* < 0.05) between corresponding treatments. **—*p* < 0.01.

The typical yellowed or wilted leaves were observed in *F. oxysporum*-inoculated CM and *spr8* plants but not in non-inoculated plants (Figure 1, Table S1). Compared with CM plants, the disease index of *spr8* plants significantly increased (*p* = 0.006 **). *R. irregularis* inoculation significantly decreased the disease index of CM plants. The control effect of *R. irregularis* on CM plants disease index was 26.67%. AM fungal inoculation had no significant effect on *F. oxysporum* development in *spr8* plants.

### 3.3. Plant Growth

**Figure 2:**
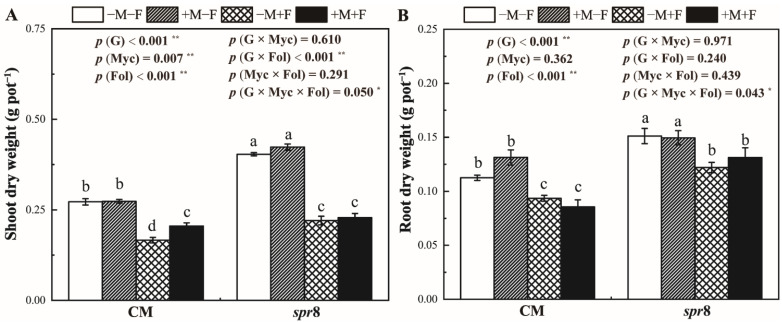
Effects of inoculation with *Rhizophagus irregularis* inoculation (Myc) and *Fusarium oxysporum* f. sp. *Lycopersici* (Fol) on the shoot (**A**) and root (**B**) dry weight of tomato CM and *spr8* plants (G). −M and +M represent non-inoculation and inoculation with *Rhizophagus irregularis*, respectively. −F and +F represent non-inoculation and inoculation with *Fusarium oxysporum* f. sp. *Lycopersici*, respectively. Same lowercase letters above the columns indicate non-significant difference (*p* < 0.05) between corresponding treatments. *—*p* < 0.05; **—*p* < 0.01.

The shoot and root dry weight of *spr8* plants were significantly higher than those of CM plants (Figure 2, Table S1). Inoculation with *F. oxysporum* significantly decreased shoot and root biomass in CM and *spr8* plants (*p* < 0.001 **). Notably, significant interactions occurred in the shoot and root biomass of plant genotypes, *R. irregularis* and *F. oxysporum* inoculation. Although inoculation with *R. irregularis* had no effect on the root dry weight, the shoot biomass of mycorrhizal CM plants was significantly higher than that of non-mycorrhizal control when inoculated with *F. oxysporum*.

### 3.4. Net Photosynthetic and Transpiration Rate

**Figure 3:**
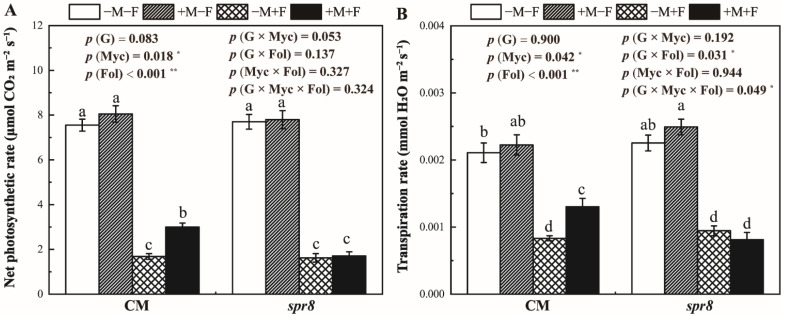
Effects of inoculation with *Rhizophagus irregularis* (Myc) and *Fusarium oxysporum* f. sp. *Lycopersici* (Fol) on the net photosynthetic rate (**A**) and transpiration rate (**B**) of CM and *spr8* plants (G). −M and +M represent non-inoculation and inoculation with *Rhizophagus irregularis*, respectively. −F and +F represent non-inoculation and inoculation with *Fusarium oxysporum* f. sp. *Lycopersici*, respectively. Same lowercase letters above the columns indicate non-significant difference (*p* < 0.05) between corresponding treatments. *—*p* < 0.05; **—*p* < 0.01.

*F. oxysporum* inoculation significantly decreased the net photosynthetic and transpiration rate of CM and *spr8* plants (Figure 3, Table S1). A three-way interaction was observed between plant genotypes, *R. irregularis* and *F. oxysporum* inoculation for transpiration rate (*p* = 0.049 *). Inoculation with *R. irregularis* significantly increased the net photosynthetic rate of CM plants under disease stress. Nonetheless, inoculation with *R. irregularis* had no significant effect on the net photosynthetic rate of *spr8* plants.

### 3.5. Shoot and Root P Concentrations

**Figure 4:**
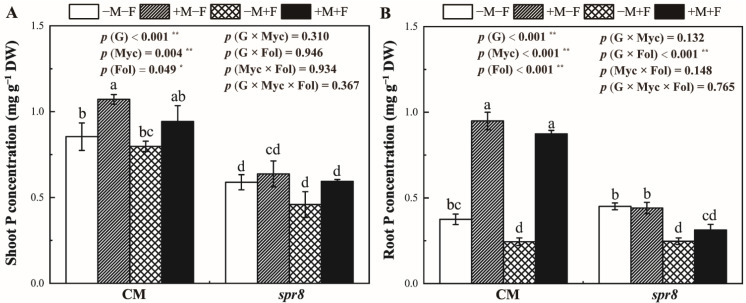
Effects of inoculation with *Rhizophagus irregularis* inoculation (Myc) and *Fusarium oxysporum* f. sp. *Lycopersici* (Fol) on the shoot (**A**) and root (**B**) P concentration of CM and *spr8* plants (G). −M and +M represent non-inoculation and inoculation with *Rhizophagus irregularis*, respectively. −F and +F represent non-inoculation and inoculation with *Fusarium oxysporum* f. sp. *Lycopersici*, respectively. Same lowercase letters above the columns indicate non-significant difference (*p* < 0.05) between corresponding treatments. *—*p* < 0.05; **—*p* < 0.01.

Inoculation with *R. irregularis* significantly increased the shoot and root P concentration of CM plants but had no effect on *spr8* plants (Figure 4, Table S2). Compared with CM −M−F, the shoot P concentration of CM +M−F increased by 25.89%. The root P concentration of CM plants showed more pronounced response to AM fungi. *F. oxysporum* inoculation decreased the root P concentration of non-mycorrhizal CM plants. No significant interactions between *R. irregularis* and *F. oxysporum* inoculation were observed on the shoot and root P concentration of both genotypes.

### 3.6. AOC and LOXD Relative Expression

**Figure 5:**
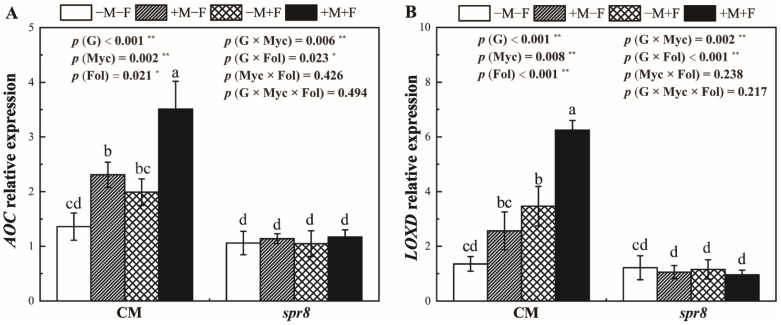
Effects of inoculation with *Rhizophagus irregularis* inoculation (Myc) and *Fusarium oxysporum* f. sp. *Lycopersici* (Fol) on the relative expression of *AOC* (**A**) and *LOXD* (**B**) of CM and *spr8* plants (G). −M and +M represent non-inoculation and inoculation with *Rhizophagus irregularis*, respectively. −F and +F represent non-inoculation and inoculation with *Fusarium oxysporum* f. sp. *Lycopersici*, respectively. Same lowercase letters above the columns indicate non-significant difference (*p* < 0.05) between corresponding treatments. *—*p* < 0.05; **—*p* < 0.01.

The relative expression of *AOC* and *LOXD* in *spr8* plants were significantly lower than those in CM plants (Figure 5, Table S2). Inoculation with *R. irregularis* or *F. oxysporum* increased *AOC* expression in CM plants and dual inoculation significantly upregulated it. The *AOC* expression of CM +M+F treatment was 158.1% higher than that of CM −M+F. Generally, the expression of *LOXD* showed a similar trend as that of *AOC* and the *LOXD* expression of CM +M+F treatment was significantly higher than that of the other treatments. No significant differences in *AOC* and *LOXD* expression in *spr8* plants were observed among different inoculation treatments.

### 3.7. PPO and PAL Activities

**Figure 6:**
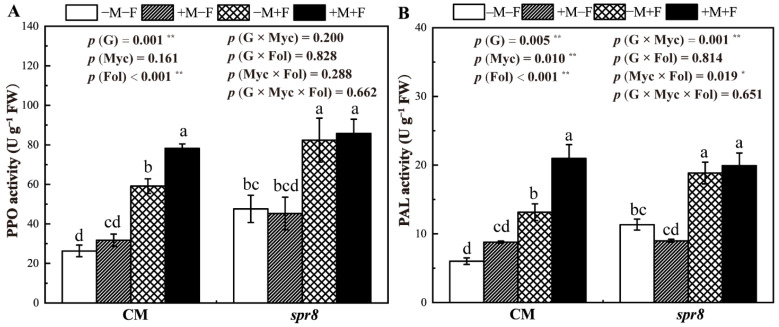
Effects of inoculation with *Rhizophagus irregularis* inoculation (Myc) and *Fusarium oxysporum* f. sp. *Lycopersici* (Fol) on the enzymes activities of polyphenol oxidase (PPO) (**A**) and phenylalnine ammonialyase (PAL) (**B**) of CM and *spr8* plants (G). −M and +M represent non-inoculation and inoculation with *Rhizophagus irregularis*, respectively. −F and +F represent non-inoculation and inoculation with *Fusarium oxysporum* f. sp. *Lycopersici*, respectively. Same lowercase letters above the columns indicate non-significant difference (*p* < 0.05) between corresponding treatments. *—*p* < 0.05; **—*p* < 0.01.

Compared with the −F controls, *F. oxysporum* inoculation significantly increased the PPO and PAL activities of CM and *spr8* plants (Figure 6, Table S2). Inoculation with *R. irregularis* significantly increased the PPO activity of CM plants inoculated with *F. oxysporum* and the PPO activity in +M plants increased by 32.20% compared with that in −M plants. Compared with CM −M+F, the PAL activities of CM +M+F increased 59.51%. There were significant interactions between plant genotypes and *R. irregularis* inoculation in PAL activities. Inoculation of *R. irregularis* had no significant effect on the PPO and PAL activities of *spr8* plants in −F and +F treatments.

## 4. Discussion

The use of AM fungi to help plants resist diseases can be a promising complementary or alternative approach to pesticide [35]. However, the effects of mycorrhiza-induced plant resistance to pathogens could depend on the AM fungus–host combinations. In the present study, we used two different tomato genotypes involving JA biosynthesis mutant (*spr8*) to determine if mycorrhizal bioprotection was genotype specific. Our experimental results demonstrated that inoculation with *R. irregularis* significantly inhibited the disease development in CM plants challenged with *F. oxysporum* but not in *spr8* plants. We also highlighted that the JA signaling pathway was highly relevant for MIR as the activated defense-related enzymes were correlated with the expression of JA biosynthesis genes in AM plants.

Mycorrhizal bioprotection effects were strongly associated with symbiosis between AM fungi and host plants [55]. The mycorrhizal colonization rate is a widely accepted key index to assess the symbiotic relationships [56]. Tomato *Spr8* is a mutant with impaired lipoxygenase D (*TomloxD*), which catalyzes the hydroperoxidation of linolenic acid [46]. *Spr8* shows a series of defective JA-mediated systemic defenses, including blocking the activity of PPO in transgenic tomato 35S::PS plants, which overexpress the *Prosystemin* gene [46]. In our experiment, the mycorrhizal colonization rates of *spr8* plants were significantly lower than those of CM plants (Table 1), confirming that the regulation of JA biosynthesis played a key role in the formation of AM symbiosis [57]. Tejeda-Sartorius et al. [58] also showed that a lack of JA synthesis significantly decreased the mycorrhizal colonization rate and arbuscule abundance of tomato plants. CM and *spr8* plants were colonized by *R. irregularis* at different levels, which may be related to the genotype-specific trait of mycorrhiza-induced protection.

*F. oxysporum* infects tomato seedlings and quickly spreads via the root vascular system, thereby interrupting water and nutrient uptake and leading to plant wilting even with sufficient water in soils [7]. Our results showed that *F. oxysporum* inoculation significantly decreased plant biomass in CM and *spr8* plants, consistent with the significantly inhibited photosynthesis (Figure 1). Notably, the disease development of *spr8* plants significantly increased, demonstrating that the *spr8* mutation impaired *F. oxysporum*-induced defenses. Yan et al. [46] also showed *spr8* plants exhibit severely compromised resistance to the necrotrophic pathogen *Botrytis cinerea*. Compared with CM plants, *spr8* plants were more susceptible to cotton bollworm (*Helicoverpa armigera*). Our results further indicated that the genetic manipulation of JA-mediated systemic defense signaling led to modified plant resistance to hemibiotrophic fungal pathogens.

AM fungi are well known to protect plants from fungal, bacterial and viral pathogens [4,24,25]. Our results showed that *R. irregularis* colonization in CM plants significantly induced bioprotection against *F. oxysporum*; however, this reduction in disease symptoms was not observed in *spr8* plants (Figure 1). The different bioprotection effects indicated that MIR was a plant genotype-specific trait. Given that plant species can largely differ in resistance induced by AM fungi against pathogens [27,59], our results suggested that plant genotypes played a pivotal role in plant–AM fungus–pathogen interaction.

Previous studies have shown that AM fungi increase plant biomass under biotic stresses as AM fungi could compensate for the damage caused by pathogenic fungi [35,60]. In this study, inoculation with *R. irregularis* significantly increased the shoot growth of CM plants inoculated with *F. oxysporum*; although, AM fungi had no significant effect on the growth of tomato plants without biotic stresses (Figure 2). AM fungi can help plants to obtain nutrients from soil, especially P [28], which further promote photosynthesis [61,62]. Our results also showed that *R. irregularis* colonization increased P content both in shoot and root (Figure 4) and increased the net photosynthetic rate (Figure 3) of CM plants. However, *R. irregularis* and *F. oxysporum* inoculation treatments showed no significant interaction on P concentration and net photosynthetic rate, indicating that the beneficial effects of *R. irregularis* was unaffected by *F. oxysporum*.

AM fungi play a critical role in plant defense activation when infected by pathogens [4,63,64]. During the establishment of mycorrhizal symbiosis, AM fungi elicit specific reactions leading to plant defense activation under biotic stresses [65,66]. AM fungi, like plant growth-promoting rhizobacteria (PGPR) and *Trichoderma* [67,68], inhibit the developments of plant pathogens through the JA signaling pathway [61] leading to defensive protein and toxin synthesis. Tian et al. [69] showed that AM fungus *Funneliformis mosseae* upregulated the expression of JA synthesis genes (*PtLOX* and *PtAOS*) in trifoliate orange (*Poncirus trifoliata*) plants infected by root rot pathogen *Phytophthora parasitica*. Pozo et al. [70] found that JA-responsive genes in mycorrhizal plants were induced to a higher level and expressed earlier. Our results showed that co-inoculation with *R. irregularis* and *F. oxysporum* significantly induced *AOC* and *LOXD* expression in CM plants; however, no significant difference was observed in *spr8* plants (Figure 5). This finding suggested that the JA signaling pathway was potentially required for MIR against *F. oxysporum.*

Generally, MIR activation upon pathogen attacks is important in plant defense responses [71]. PPO is the key enzyme in the oxidation of polyphenols to quinons, which are antimicrobial compounds, whereas PAL is involved in phenylpropanoid metabolism, which is closely related to the synthesis and accumulation of phenols, lignin and antitoxin [72]. PPO and PAL participate in the defense reaction by inducing plant resistance against pathogenic fungi [25] as these enzymes contribute to the induced resistance against *F. oxysporum* f.sp. *cubense* in bananas [73]. The increase of defense enzymes was closely related to JA [74], which can enhance plant resistance by activating defense-related enzymes such as PPO and POX [75]. Zhang et al. [76] confirmed that JA is a central player in PPO-mediated tea resistance against tea geometrids. Campos-Vargas et al. [77] found that JA can elevate PAL activity in lettuce plants. In the present study, the PPO and PAL of plants infected by *F. oxysporum* are significantly upregulated, confirming that these are marker enzymes that could be induced by plant pathogens. Increased PPO and PAL activities were recorded in CM plants co-inoculated with *R. irregularis* and *F. oxysporum*, demonstrating the activation of MIR for protecting plants against these soilborne pathogens. However, contrasting results of AM fungi on the PPO and PAL activities of *spr8* plants have been reported, which can be explained by the fact that the efficient MIR relies on a functional JA signaling pathway.

## 5. Conclusions

This experiment explored and clarified the bioprotection effects and mechanisms of AM symbiosis against *Fusarium* wilt using the tomato JA biosynthesis defective mutant. Results demonstrated that the disease protection of AM fungi was a plant genotype-specific trait as inoculation with *R. irregularis* significantly reduced the disease index of CM plants but not that of *spr8* plants. Importantly, our results further showed that *R. irregularis* colonization upregulated JA synthesis gene expression in CM plants, leading to enhanced resistance against *F. oxysporum* with increased PPO and PAL activities. Thus, the JA signaling pathway was highly relevant to MIR against *F. oxysporum*. The study confirmed that AM fungi can potentially server as biological control agents for the management of *Fusarium* wilt disease.

## Figures and Tables

**Table 1:** Effects of inoculation with *Rhizophagus irregularis* inoculation (Myc) and *Fusarium oxysporum* f. sp. *Lycopersici* (Fol) on the mycorrhizal colonization rate and arbuscule abundance of CM and *spr8* plants (G). −M and +M represent non-inoculation and inoculation with *Rhizophagus irregularis*, respectively. −F and +F represent non-inoculation and inoculation with *Fusarium oxysporum* f. sp. *Lycopersici*, respectively. Same lowercase letters after the values in the same column indicate non-significant difference (*p* < 0.05) between corresponding treatments. *—*p* < 0.05; **—*p* < 0.01.

Genotypes	Inoculation	Mycorrhizal Colonization Rate (%)	Arbuscule Abundance (%)
CM	−M−F	0.0 ± 0.0 d	0.0 ± 0.0 c
	+M−F	58.8 ± 4.0 a	33.9 ± 4.4 a
	−M+F	0.0 ± 0.0 d	0.0 ± 0.0 c
	+M+F	50.5 ± 3.1 b	38.1 ± 1.6 a
*Spr8*	−M−F	0.0 ± 0.0 d	0.0 ± 0.0 c
	+M−F	31.6 ± 3.5 c	13.7 ± 2.1 b
	−M+F	0.0 ± 0.0 d	0.0 ± 0.0 c
	+M+F	32.7±1.4 c	11.4 ± 1.6 b
**Significance of**			
G		*p* < 0.001 **	*p* < 0.001 **
Myc		*p* < 0.001 **	*p* < 0.001 **
Fol		*p* = 0.219	*p* = 0.861
G × Myc		*p* < 0.001 **	*p* < 0.001 **
G × Fol		*p* = 0.163	*p* = 0.229
Myc × Fol		*p* = 0.219	*p* = 0.861
G × Myc × Fol		*p* = 0.163	*p* = 0.229

## Data Availability

The datasets generated during the current study are available from the corresponding author on reasonable request.

## Supplementary Materials

The following supporting information can be downloaded at: https://www.mdpi.com/article/10.3390/jof8050422/s1, Table S1: Means ± SE of data shown in Figure 1, Figure 2 and Figure 3; Table S2: Means ± SE of data shown in Figure 4, Figure 5 and Figure 6.
