# Supplementary material for: Arbuscular Mycorrhizal Fungi Induced Plant Resistance against Fusarium Wilt in Jasmonate Biosynthesis Defective Mutant and Wild Type of Tomato
Source: J Fungi (Basel). 2022 Apr 20;8(5):422. doi: 10.3390/jof8050422 (PMC9146357; doi:10.3390/jof8050422)
Supplement: Supplementary file 1 [file jof-08-00422-s001.zip › jof-1654405-supplementary.pdf]

Supplementary Table S1 Means±SE of data shown in Figures 1-3. −M and +M represent non-inoculation and inoculation with *Rhizophagus irregularis*, respectively. −F and +F represent non-inoculation and inoculation with *Fusarium oxysporum* f. sp. *Lycopersici*, respectively.

Genotypes	Inoculation	Disease index	Shoot dry weight (mg pot^{-1})	Root dry weight (mg pot^{-1})	Net photosynthetic rate (μmol CO_2 $m^{-2}s^{-1}$)	Transpiration rate (μmol H_2O $m^{-2}s^{-1}$)
CM	−M−F	0.00±0.00	272.13±8.67	115.27±2.44	7.55±0.29	2.108±0.154
	+M−F	0.00±0.00	273.58±5.05	131.33±6.98	8.05±0.39	2.308±0.154
	−M+F	41.67±4.81	166.40±7.64	93.50±2.79	1.68±0.14	0.833±0.039
	+M+F	30.56±2.78	205.40±8.94	85.51±6.55	3.00±0.20	1.304±0.137
Spr8	−M−F	0.00±0.00	403.58±4.49	151.20±7.04	7.70±0.35	2.254±0.125
	+M−F	0.00±0.00	423.22±8.65	149.49±5.05	7.80±0.43	2.493±0.124
	−M+F	50.00±4.81	220.42±11.88	122.00±4.76	1.62±0.20	0.945±0.078
	+M+F	47.22±2.78	228.66±11.25	131.17±9.10	1.71±0.19	0.813±0.114

Supplementary Table S2 Means±SE of data shown in Figures 4-6. −M and +M represent non-inoculation and inoculation with *Rhizophagus irregularis*, respectively. −F and +F represent non-inoculation and inoculation with *Fusarium oxysporum* f. sp. *Lycopersici*, respectively.

Genotypes	Inoculation	Shoot P concentration (mg g^{-1} DW)	Root P concentration (mg g^{-1} DW)	*AOC* relative expression	*LOXD* relative expression	PPO activity (U g^{-1} FW）	PAL activity (U g^{-1} FW）
CM	−M−F	0.85±0.08	0.37±0.03	1.36±0.25	1.36±0.27	26.33±2.95	6.02±0.47
	+M−F	1.07±0.03	0.95±0.05	2.31±0.23	2.57±0.69	31.76±3.07	8.80±0.16
	−M+F	0.80±0.03	0.25±0.02	1.99±0.24	3.47±0.72	59.14±3.68	13.15±1.22
	+M+F	0.94±0.09	0.87±0.02	3.51±0.51	6.25±0.35	78.22±2.20	20.97±2.01
Spr8	−M−F	0.59±0.04	0.45±0.02	1.06±0.22	1.22±0.44	47.58±6.89	11.34±0.80
	+M−F	0.64±0.08	0.44±0.03	1.14±0.09	1.06±0.24	45.27±8.24	8.99±0.22
	−M+F	0.46±0.07	0.25±0.02	1.05±0.23	1.16±0.35	82.38±11.14	18.85±1.59
	+M+F	0.59±0.01	0.31±0.03	1.17±0.13	0.96±0.17	85.79±7.15	19.94±1.82
